# Supplementary material for: eYGFPuv-Assisted Transgenic Selection in Populus deltoides WV94 and Multiplex Genome Editing in Protoplasts of P. trichocarpa × P. deltoides Clone ‘52-225’
Source: Plants (Basel). 2023 Apr 14;12(8):1657. doi: 10.3390/plants12081657 (PMC10145771; doi:10.3390/plants12081657)
Supplement: Supplementary file 1 [file plants-12-01657-s001.zip › plants-2121311-supplementary.pdf]

Supplementary Materials

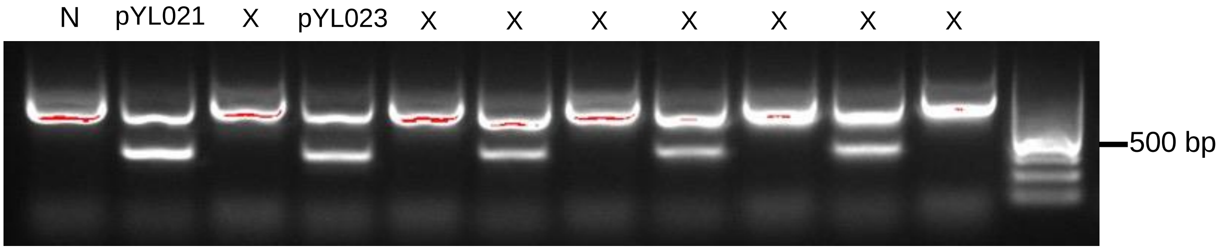

Figure S1. Amplicons of the *phytoene desaturase* (*PDS*) target region from the 52-225 protoplast cells transformed by pYL021 and pYL023. N indicates negative control transformed with pGFPGUSPlus. x indicates the products are not relevant to this study.

Table S1. Plasmids used and constructed in this study.

**Name**	**Description**	**References**
eYGFPuv/GUS	Dual reporter vector	[1]
GFPGUSplus	Dual reporter vector	[2]
pAXY0003	Dual reporter vector	This study
pMOD_A0902	TREX2-P2A-AtCas9 driven by AtUbi10	[3]
pMOD_B0000	Empty module B vector	[3]
pMOD_C0000	Empty module C vector	[3]
pTRANS_220d	T-DNA backbone	[3]
pTRANS_221	T-DNA backbone with BeYDV replicon.	[3]
pYL009	Module C: GFPuv expression vector	This study
pYL015	Module B: gRNAs expression vector	This study
pYL021	Gene editing T-DNA vector without replicon for targeting PDS	This study
pYL023	Gene editing T-DNA vector with replicon for targeting PDS	This study

Table S2. Primers used in this study.

Primer name	Primer sequence (5′-3′)
Genotype_F	CACGGCAACCTCAACG
Genotype_R	CTCGACACGTCTGTGGG
oYL_063_Deep_seq_F	GTTGAATTTGGTTTTGGAGAAATG
oYL_062_Deep_seq_R	TTGCAGTCGATAAACCCGCC

References:

1. Yuan, G.L.; Lu, H.W.; Tang, D.; Hassan, M.M.; Li, Y.; Chen, J.G.; Tuskan, G.A.; Yang, X.H. Expanding the application of a UV-visible reporter for transient gene expression and stable transformation in plants. *Hortic Res-England* **2021**, *8*, 234.
2. Vickers, C.E.; Schenk, P.M.; Li, D.; Mullineaux, P.M.; Gresshoff, P.M. pGFPGUSPlus, a new binary vector for gene expression studies and optimising transformation systems in plants. *Biotechnol Lett* **2007**, *29*, 1793-1796.
3. Cermak, T.; Curtin, S.J.; Gil-Humanes, J.; Cegan, R.; Kono, T.J.Y.; Konecna, E.; Belanto, J.J.; Starker, C.G.; Mathre, J.W.; Greenstein, R.L.; et al. A Multipurpose Toolkit to Enable Advanced Genome Engineering in Plants. *Plant Cell* **2017**, *29*, 1196-1217.
